# Lessons From Building a Large, Public HIV-Related Database in Support of the Ending the HIV Epidemic in the US Initiative

**DOI:** 10.2196/72088

**Published:** 2026-07-27

**Authors:** Victor Wang, Nikita Rao, Sheree R Schwartz, Sten H Vermund, Lien Quach, Rafael Perello Carbonell, Artur Queiroz, Kush Shanker, Sung-Jae Lee, Joyce L Jones, Debbie Humphries, Donna Spiegelman

**Affiliations:** 1 Center for Methods in Implementation and Prevention Science Yale School of Public Health New Haven, CT United States; 2 Division of Infectious Disease Epidemiology Department of Epidemiology Johns Hopkins University Baltimore, MD United States; 3 Institute for Sexual and Gender Minority Health and Wellbeing Northwestern University Evanston, IL United States; 4 Department of Psychiatry and Biobehavioral Sciences University of California, Los Angeles Los Angeles, CA United States; 5 Department of Medicine Johns Hopkins University Baltimore, MD United States

**Keywords:** HIV, data integration, Ryan White, implementation science, Ending the HIV Epidemic, social determinants of health, public health databases

## Abstract

The HIV epidemic remains a national priority in the United States, and the Ending the HIV Epidemic initiative has renewed the call for expanded prevention and treatment strategies capable of reducing new HIV infections by 90% by 2030. Achieving this goal requires robust, integrated data for understanding HIV-related needs, barriers to care, and the effectiveness of interventions. However, despite the existence of numerous publicly available datasets, few integrate multiple domains such as HIV outcomes, social determinants of health, and community-level factors. The lack of unified data and difficulty linking datasets hampers efforts for meaningful cross-domain analyses to tailor HIV management and treatment strategies. The resulting fragmentation constitutes a methodological gap: implementation teams lack replicable guidance for constructing unified HIV and contextual databases from public sources. In this viewpoint, we describe our experience building a unified compilation of publicly available HIV and community data to identify factors influencing HIV outcomes and interventions. The completed database comprises 242 variables drawn from 8 public sources mapped across clinic, zip code, county, and state levels of geography. Rather than simply reporting what we built, we position four core decisions as transferable methodological advances: (1) treating source identification as a bounded phase before construction begins, (2) adopting automated data engineering tools from the outset rather than manual entry, (3) establishing a shared data dictionary before the first variable is entered, and (4) integrating quality control throughout the workflow rather than as a final phase. The build required approximately 350 total project hours and revealed an initial spot-check error rate of approximately 33%, which we attribute primarily to manual data entry. By sharing the approach used to develop this database and making the final resource publicly accessible through the Yale Center for Methods in Implementation and Prevention Science, we aim to reduce barriers to data access and encourage similar data integration efforts. The methodological framework described in this paper is intentionally designed to be replicable with modest resources, and we present it as a practical model for research teams operating without specialized infrastructure. Consolidating HIV, social determinants of health, and contextual variables into a unified data source is a critical step toward enabling deeper, more comprehensive analysis and supporting ongoing efforts to end the HIV epidemic in the US.

## Introduction

The Ending the HIV Epidemic (EHE) initiative is a national priority involving multiple government and health care organizations across the United States. Despite meaningful progress, HIV transmission and care retention remain below the goal of reducing new HIV infections by 90% by 2030 [1-5]. A critical and underappreciated barrier to achieving this goal is the fragmented data infrastructure underlying implementation efforts: public datasets covering HIV outcomes, social determinants of health (SDOH), and community context exist but are rarely linked in ways that allow for meaningful cross-analysis to tailor HIV care and improve access to HIV treatment and prevention services. The result is an analytic blind spot where implementation decisions are made [6,7].

Large, integrated datasets have proven their value in HIV research, such as for understanding preexposure prophylaxis barriers [8] and evaluating intervention outcomes and for analogous infectious disease contexts such as tuberculosis [9]. However, building such databases from public sources is rarely described in enough detail to be replicated. Researchers recognize that integrating data tools would be useful, but they have little practical guidance on how to construct them efficiently and reliably. This viewpoint addresses that gap directly.

As part of a larger multisite study effort aimed at informing strategies to scale up the implementation of rapid, status-neutral antiretroviral therapy for treatment and prevention within and across 6 EHE priority jurisdictions in the United States [10-12], our team built a linked HIV and contextual database using publicly available data. We argue that this kind of build is achievable with modest resources, and we present our experience not as a project report but as a structured set of transferable methodological lessons framed around the decisions that most shaped the process, errors that proved most instructive, and practices that future teams should adopt from the onset.

### Database Development

#### Overview

**Figure 1:**
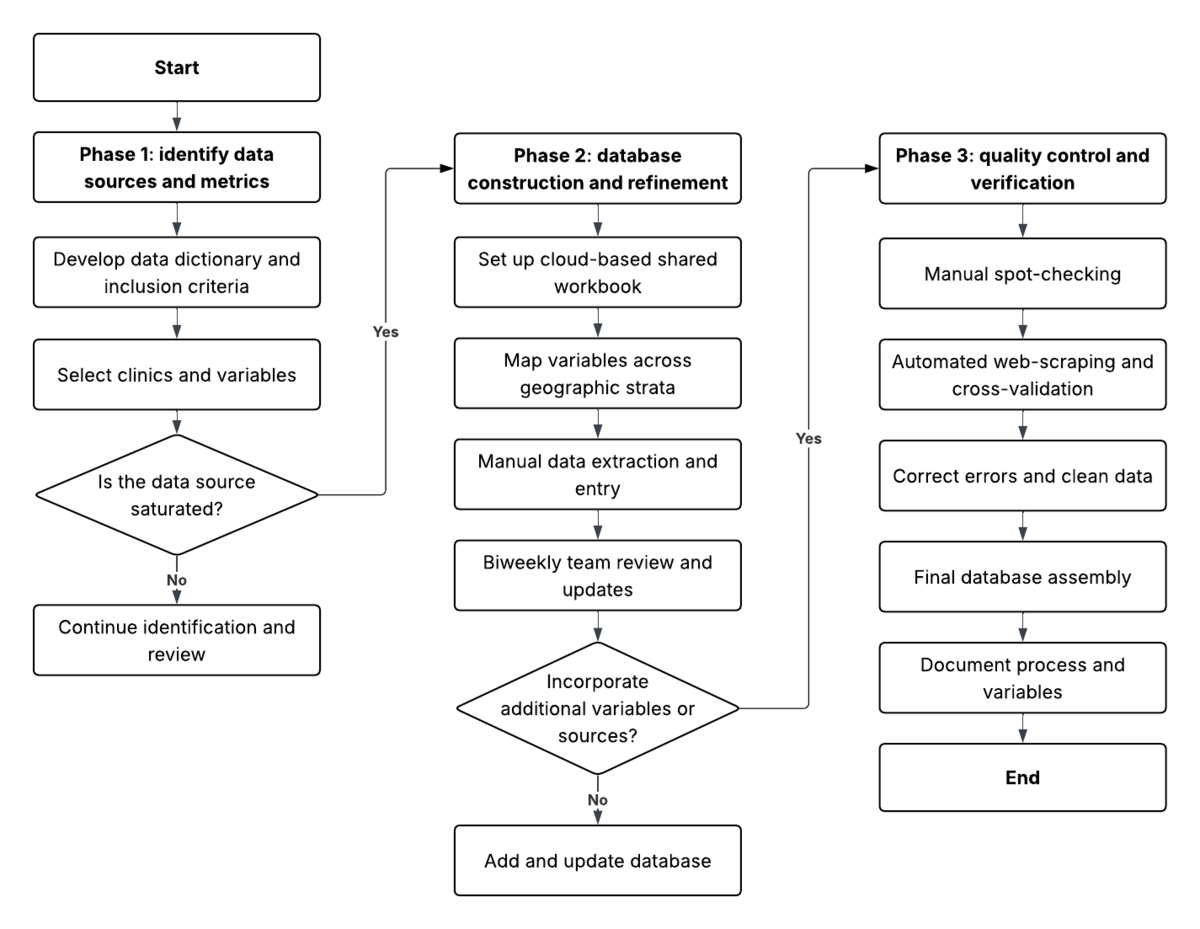
Process flow for data extraction and cleaning.

Drawing on our experience developing the database (Figure 1), we identified 4 key methodological principles for harmonizing public HIV and SDOH data. These findings carry direct implications for how future teams should structure similar work.

#### The Database: What We Built

The final database comprises 242 variables pulled from 8 publicly available data sources (Table 1) supplemented by 14 clinic-specific variables (eg, Ryan White HIV/AIDS Program parts, clinic address, clinic name, and associated HIV Implementation Science Coordination Initiative hub) made available by investigators participating in the parent multisite study. The 104 Ryan White HIV/AIDS Program–associated clinics of interest, the primary unit of analysis, were located in 5 states: Maryland, Illinois, Alabama, Texas, and California (Table 2) [13]. Variables were mapped across various levels of geography: state, county, zip code, and individual clinic [14]. The HIV Implementation Science Coordination Initiative serves as a National Institutes of Health–funded technical support network that enhances the use of implementation science and related qualitative and quantitative methods in National Institutes of Health–funded EHE pilot projects [15].

**Table 1:** Table of data sources included in the dataset.

Data source	Description	Geographic level	Variables included in the final dataset, n	Data extraction method	Link
US census 2020	Population demographics and socioeconomic status	Zip code^a^ and county	32	Manual extraction	[16,17]
Kaiser Family Foundation	Syringe exchange programs, HIV outcomes, life expectancies, and Medicaid expansion year	State	7	Manual extraction	[18]
The Center for HIV Law and Policy	HIV criminalization laws	State	1	Manual extraction	[19]
AIDSVu	Demographics of patients with HIV, treatment and prevention need, and transmission rates	County	58	Manual extraction	[20]
University of Wisconsin Population Health Institute County Health Rankings	Health outcomes, socioeconomic status, HIV rates, comorbidities, insurance rates, preventative care rates, school funding, crime, and access to services	County	70	Manual extraction	[21]
CDC^b^	AIDS and HIV rates, treatments, outcomes, and socioeconomic status	State and county	18	Manual extraction	[22]
HRSA^c^	Health outcomes, demographics, and clinic costs	Clinic	69	Manual extraction	[23]
Walk Score	Walkability	Zip code	1	Manual extraction	[24]
Ryan-White clinic survey	Private clinic-level data	Clinic	26	Private data from the multisite study	[10]

^a^Zip code refers to a US postal code system for mail delivery and geographic identification.

^b^CDC: Centers for Disease Control and Prevention.

^c^HRSA: Health Resources and Services Administration.

Of the 242 variables, 238 (98.3%) were continuous variables (eg, percentages and rates), consistent with the nature of the data (eg, social determinants and population statistics), and 4 (1.7%) were categorical variables (eg, geographic coding for HIV-related laws, urban and rural classification, and Safe Drinking Water Act violations). Variables were grouped into four conceptual domains (Tables S1-S3 in Multimedia Appendix 1): (1) environmental variables (eg, population demographics, employment, poverty, housing, education, health care access, and crime), (2) HIV-related variables (eg, demographics of people living with HIV, HIV prevalence and incidence, HIV criminalization laws, and infection routes), (3) implementation outcome variables (eg, HIV mortality rates, preexposure prophylaxis use, and viral suppression), and (4) clinic variables (eg, patient demographics and patient financial information).

The dataset, data dictionary, and SAS code are publicly available at the Yale Center for Methods in Implementation and Prevention Science website [25]. The data dictionary maps each variable to its definition, SAS label, source, data type, and geographic level and is a resource we consider as important as the database itself.

**Table 2:** Number of clinics by county ordered by state (N=104).

State and county			Clinics, n
**AL^a^**			
	Calhoun County	1	
	Jefferson County	2	
	Lee County	1	
	Madison County	1	
	Mobile County	3	
	Montgomery County	1	
	Tuscaloosa County	1	
**CA^b^**			
	Los Angeles County	33	
	Orange County	1	
	San Diego County	14	
**IL^c^**			
	Cook County	22	
	St Clair County	1	
**MD^d^**			
	Baltimore City	18	
**TX^e^**			
	Dallas County	1	
	Tarrant County	4	

^a^AL: Alabama.

^b^CA: California.

^c^IL: Illinois.

^d^MD: Maryland.

^e^TX: Texas.

### Lessons Learned

#### Overview

The database build unfolded across 3 phases: source identification (approximately 2 months), database construction (approximately 6 months), and quality control (approximately 6 weeks), requiring approximately 350 total hours across 3 team members (roughly 250 core build hours for data sourcing, quality control, and cleaning plus approximately 100 hours for meetings and project management). What follows is not a chronological project account but a distillation of the 4 decisions that most shaped our experience, with each framed explicitly as a transferable methodological advance. These lessons are grounded in prior literature in public health data integration, which consistently highlights that successful integration depends on interoperable, standard-based systems such as Fast Healthcare Interoperability Resources [26], which facilitate efficient data exchange between health care and public health systems. Federal initiatives such as the Centers for Disease Control and Prevention’s Data Modernization Initiative and the Trusted Exchange Framework and Common Agreement underscore the value of standardized electronic case reporting to enhance the timeliness and completeness of public health data [27,28]. Research on chronic disease surveillance further highlights ongoing fragmentation across data sources and calls for integrated, multi-sectoral data pipelines to reduce silos and improve data quality [29]. Our experience both confirms and extends these findings by providing a concrete, replicable methodology for building such pipelines from publicly available sources.

#### Lesson 1: Treat Source Data as a Bounded, Saturation-Driven Phase

One of our most consequential decisions was spending 2 months identifying and vetting sources prior to database construction. As we engaged in this process, key data sources and variables emerged through expert consultations and iterative review until no new sources emerged and saturation was reached. Selection criteria prioritized relevance to project goals and data availability. Clinic inclusion was limited to clinics participating in the parent multisite study with available geographic data. The diversity of and open access to public data were critical in supporting database development. The existence of datasets that covered a range of themes and were tagged to geographic levels made pulling and linking datasets by geography simple and feasible. Furthermore, many datasets allowed for direct download, facilitating linkage and checking. Notably, some datasets were already preaggregated for the purpose of supporting analysis of HIV determinants, which limited the number of unique sources to be identified. We recommend this approach strongly, with one caveat: source identification should be treated as a discrete phase. As we lacked a centralized repository of HIV and SDOH datasets, sources were sometimes added ad hoc as expert input was received throughout the database build. This pattern forced us to repeatedly revisit and update things that were already built, which led to complicated version control. Teams should treat source identification as a bounded phase with clear saturation criteria and resist adding sources once construction has begun. To facilitate consistency and continuity, a comprehensive data dictionary was developed early in this phase. This served as a foundational reference for both existing and new team members by outlining each variable’s definition, source, data type, and geographic level. We were mindful of potential variable selection bias and attempted to minimize it by applying clear inclusion criteria aligned with study objectives and documenting all decisions within the data dictionary. During this phase, preliminary metrics were cataloged in an offline Microsoft Excel workbook before being transferred to a cloud-based Microsoft Excel platform to enable collaborative integration. This step proved essential for coordinating work across team members and maintaining a shared understanding of emerging data structures, especially for onboarding new contributors. We regard this documentation step as nonnegotiable.

#### Lesson 2: Adopt Automated Data Engineering Tools From the Outset

This is our most direct recommendation. Our database build relied primarily on manual data entry, a method that, while adaptable, resulted in a notable initial error rate of approximately 33%, computed as flagged discrepancies divided by total spot-checked values (roughly 10% of all entered data, sampled as every fifth row per source and geographic level). An “error” was defined as any value that differed from the authoritative source on the date of verification, including discrepancies from data entry mistakes, source updates, or rounding differences. All flagged discrepancies were subsequently corrected, but the rework was substantial.

This experience highlights an important limitation and area for process-related improvement. Future efforts would benefit from the adoption of standard data engineering tools and automation, such as SQL databases; extract, transform, and load pipelines; application programming interfaces, and web scraping libraries such as R’s *rvest* [30] or Python’s pandas [31]. We adopted automated web scraping later in the build for quality control, which substantially reduced reconciliation effort and would have reduced errors had it been used for initial extraction. While there is a learning curve for these tools, their integration could enhance data accuracy, reduce labor-intensive manual processes, and improve overall efficiency in database construction and maintenance.

We also recommend using a version-controlled, access-managed cloud-based platform (we used Microsoft Excel hosted on Northwestern University’s secure servers). This approach ensured that all team members accessed a single, up-to-date version of the database. Asynchronous communication, including email, enabled the team to divide tasks across different time zones and coordinate effectively but only because version control prevented conflicts that often arise with parallel local editing.

#### Lesson 3: Build Quality Control Into Every Phase of the Workflow

In our build, quality control was only applied in the final phase. We recommend that it be built into the workflow of the entire database construction in future builds. While we relied on 2 complementary approaches that helped us assess the consistency and accuracy of the compiled data (manual spot checks and use of a third-party web scraping tool), errors had accumulated over months of entry. The 33% discrepancy rate we observed reflects not only the error-prone nature of manual data entry, but equally the decision to conduct validation and quality checks only after months of data entry had occurred rather than building them in continuously throughout the database development process.

We recommend integrating verification at each data entry milestone: upon completing each source, each geographic level, and each domain. Other practical considerations for future database builds include the importance of documenting decisions, establishing shared naming conventions and data format standards before entry begins, and incorporating tools not only for quality control but also for initial extraction where possible.

#### Lesson 4: Small, Stable Teams Are Effective When Timelines Are Protected

Although several experts were involved in identifying databases, the final build was primarily executed by 1 Master’s student under the guidance of a lead researcher, with 3 team members contributing to data extraction. This lean structure enabled efficient communication and clear accountability. We found that expanding the team would have introduced coordination costs that likely exceeded the time saved.

However, team stability depends on protected time. One direct consequence of resource constraints was that the clinic sample had to be fixed before the parent multisite study was finalized, resulting in a smaller clinic count than the number ultimately enrolled in the main study. Teams should anticipate that timeline pressures create scope constraints and plan accordingly. While there is potential to improve efficiency, the feasibility of this approach suggests that similar builds can be accomplished with modest teams and resources if timelines and availability are managed effectively. We estimate that a team with this structure can complete a comparable build in roughly 250 to 350 hours with 250 core build hours (data sourcing, quality control, and cleaning) plus approximately 100 hours of meetings and project management, although automated tooling from the start could reduce the core build hours substantially.

### Key Observations

Despite the process challenges, the build succeeded. The resulting database covers the breadth of variables needed to support EHE-relevant analysis: 238 continuous variables and 4 categorical variables spanning environmental context, HIV epidemiology, implementation outcomes, and clinic-level characteristics. Missingness was low overall, although some zip codes, states, and clinics lacked specific variables; this limitation is noted in the “Missing, %” column of the descriptive tables in Multimedia Appendix 1 and addressed through proxy exploration where feasible.

The dataset demonstrates that clinic-specific, geographically linked HIV databases are feasible to construct from public sources [32,33]. Our build required no proprietary data access and no specialized computational infrastructure. The resources required—roughly 250 core build hours and a small, coordinated team—are within reach of most academic research groups. The fully documented database and SAS processing code are available at the Yale Center for Methods in Implementation and Prevention Science website [25], and we encourage reuse, extension, and critique.

The creation of the database holds tremendous value in both understanding processes with respect to building and hosting large databases and in advancing the goals of the EHE initiative. This effort illustrated the feasibility of such a build, requiring approximately 350 hours of effort and involving an initial error rate of 33%, highlighting both the time investment and quality control needs involved. An example of a potential use of the database could be for exploratory analysis of HIV-related metrics to identify factors that drive HIV transmission or barriers to care [6,7]. Another use could be in assessing congruence and correlations across datasets that capture similar metrics. Additionally, the database could be instrumental in tracking progress toward EHE goals both for HIV-specific outcomes (such as HIV prevalence, testing, and treatment rates) and for secondary effects, including changes in sexually transmitted disease transmission rates, access to care, and neighborhood poverty levels [34,35]. This dual focus on HIV-related outcomes and broader social and health metrics creates valuable opportunities for holistic public health analysis [36,37].

Given the database’s reliance on publicly available datasets, it is important to acknowledge the growing uncertainty regarding sustained access to such data. Recent trends toward increased data protection and tightening access policies may challenge the long-term reproducibility of similar public health infrastructure efforts. This underscores the importance of archiving and sharing harmonized datasets wherever possible to preserve transparency and support future applications.

### Ethical Considerations

This study used only publicly available, deidentified data and did not involve human subject research. It was deemed exempt from review by the Yale University Institutional Review Board under its Human Research Protection Program policy for secondary research using public data [38]. No personal identifiable information or individual-level data were used. No images or materials depicting identifiable individuals are included in this manuscript or its supplementary files.

### Conclusions and Future Directions

In supporting the EHE initiative, this collaborative project addressed a critical gap in unified data and the ability to perform comprehensive analyses across a range of thematic variables, including geographic disparities [39], SDOH [40], and individual characteristics. This challenge hampers our ability to understand the pertinent factors associated with HIV need, which geographies are most impacted, what SDOH indicators are critical in addressing variations in HIV outcomes, and what interventions may be most effective for specific communities [3,41].

Given our goal of also providing the database as a publicly available dataset, there is significant potential for future expansion. Additional datasets, both HIV specific and related to other public health domains, could be integrated into the database. The inclusion of individual-level data and more comprehensive SDOH indicators would provide even greater granularity, allowing for deeper insights into the complex factors influencing HIV-related outcomes [35-37]. Furthermore, the database could serve as a platform for cross-referencing and corroborating data across a range of metrics, opening up new opportunities for exploring key questions and informing public health policy. Several datasets, some of which require requests for access, were identified during this project and are outlined in Multimedia Appendix 1 for future consideration. In addition, descriptive tables of the variables organized according to clinic variables, health outcome variables, implementation outcome variables, and environmental variables are also included in Multimedia Appendix 1. These materials can serve as a foundation for expanding the database in the future and further advancing the goals of the EHE initiative. We anticipate expanding the dataset beyond the initial 104 clinics to improve utility and generalizability as resources and data access permit.

Looking ahead, data scraping practices will be a key consideration to enhance the efficiency and accuracy of data collection. R-based tools such as the *rvest* package [30] could be prioritized for web scraping, offering a more flexible and efficient solution for extracting data. This may help streamline the process and improve scalability, particularly when scraping large and diverse datasets. Adopting best practices (eg, setting up error handling and ensuring legal compliance) helps minimize issues related to incomplete or inaccurate data. Moreover, using structured workflows for scraping helps ensure that data are collected in a systematic and consistent manner, integrating data into the database while supporting quality control processes. These enhancements can help improve the sustainability of the database and facilitate future updates as new data sources emerge, further supporting the goals of the EHE initiative. Our viewpoint is that the field does not need to wait for infrastructure to do useful integration work. Both public datasets and the tools to link them are available now. What has been missing is a practical, experience-based account of how to do this work efficiently. We offer this account as a replicable model, with the explicit expectation that future teams will improve upon it. Building on this experience, our team has already begun developing an additional database applying the lessons learned from this project to strengthen harmonization, improve workflow efficiency, and broaden the scope of integrated public health data resources.

## Data Availability

The datasets generated or analyzed during this study are available in the Center for Methods in Implementation and Prevention Science repository [42].

## Appendix

Multimedia Appendix 1 Descriptive tables.

## Abbreviations

CFAR: Centers for AIDS Research
EHE: Ending the HIV Epidemic
SDOH: social determinants of health
