# Supplementary material for: Lessons From Building a Large, Public HIV-Related Database in Support of the Ending the HIV Epidemic in the US Initiative
Source: JMIR Form Res. 2026 Jul 27;10:e72088. doi: 10.2196/72088 (PMC13458351; doi:10.2196/72088)
Supplement: Supplementary file 1 [file formative_v10i1e72088_app1.docx]

**Appendix: Descriptive Tables**

| **Table S-1** *Environmental Variables (Demographics, Social Environment of People Living with HIV/AIDS, Healthcare Access, and Resources)^[1]^* | | |  |
| --- | --- | --- | --- |
|  | Mean (SD) or % (SD)  (n = 104) | Missing, % |  |
| **Population** |  |  |  |
| Estimated population size (zipc), 2020, (Mean (SD) | 4930877  (3742123) | 0.0 |  |
| Female (zipc), 2020 (%) | 51.1 (1.2) | 1.9 |  |
| Density (persons/mi2) (cnty), 2020 | 3464 (2366) | 0.0 |  |
| **Rural / Urban Communities** |  |  |  |
| Rural population (clinic state), 2019 (%) | 11.7 (10.3) | 0.0 |  |
| County classification as urban (clinic cnty), 2019, % | 100.0 | 0.0 |  |
|  |  |  |  |
| CDC ranking of level of urbanicity of county (clinic cnty), 2020, % | 100.0 | 9.6 |  |
| - Large Urban, % |  |  |  |
| **Age** |  |  |  |
| <5 (zipc), 2020 (%) | 5.7 (0.4) | 1.9 |  |
| <18 (zipc), 2020 (%) | 21.5 (1.2) | 1.9 |  |
| 18-65 (zipc), 2020 (%) | 64.3 (5.2) | 0.0 |  |
| >65 (zipc), 2020 (%) | 15.0 (1.0) | 1.9 |  |
| **Race** |  |  |  |
| Asian (zipc), 2020 (%) | 9.7 (5.5) | 1.9 |  |
| White (zipc), 2020 (%) | 61.9 (15.0) | 1.9 |  |
| Non-Hispanic White (zipc), 2020 (%) | 35.6 (11.2) | 1.9 |  |
| Black (zipc), 2020 (%) | 24.2 (20.4) | 1.9 |  |
| American Indian (zipc), 2020 (%) | 1.0 (0.4) | 1.9 |  |
| Native Hawaiian or Pacific Islander (zipc), 2020 (%) | 0.3 (0.2) | 4.8 |  |
| Hispanic (zipc), 2020 (%) | 29.1 (17.4) | 1.9 |  |
| Multiracial (zipc), 2020 (%) | 3.0 (0.9) | 1.9 |  |
| **Family Dynamics** |  |  |  |
| Mean family size (zipc), 2020 | 3.3 (0.5) | 8.7 |  |
| Have children (zipc), 2021 (%) | 18.9 (7.6) | 5.8 |  |
| Childcare costs for a household with two children as a percent of median household income (cnty), 2020-21 (%) | 28.2 (5.1) | 0.0 |  |
| Children living in single-parent household (cnty), 2016-20 (%) | 31.0 (10.1) | 0.0 |  |
| Never married (zipc), 2020 (%) | 49.6 (14.1) | 5.8 |  |
| Number of organizational memberships per 10K population^[2]^ (cnty), 2019 | 7.6 (2.2) | 0.0 |  |
| Teen births (per 1K females 15-19) (cnty), 2014-20 | 20.5 (7.0) | 0.0 |  |
| **Income** |  |  |  |
| Median county income ($) (zipc), 2020 | 37959.0 (3956.4) | 1.9 |  |
| Median household income ($) (cnty), 2020 | 70027.9 (11977.9) | 0.0 |  |
| Ratio of female to male median earnings for full-time workers (cnty), 2016-20 | 0.9 (0.1) | 0.0 |  |
| Ratio of 80th to 20th income percentile (cnty), 2016-20 | 5.4 (0.5) | 1.0 |  |
| Income inequality (GINI) coefficient (0.0-1.0) (cnty), 2020 | 0.5 (0.0) | 10.6 |  |
| Hourly wage needed for basic expenses and taxes for an adult and two children ($) (cnty), 2021 | 44.5 (5.4) | 0.0 |  |
| **Poverty** |  |  |  |
| Poverty (cnty), 2020 (%) | 13.8 (3.5) | 10.6 |  |
| <18 and in poverty (cnty), 2020 (%) | 18.3 (5.1) | 0.0 |  |
| Households below poverty level (state), 2019 (%) | 10.8 (2.4) | 0.0 |  |
| Low-income and living far from grocery stores (cnty), 2019 (%) | 3.0 (3.2) | 0.0 |  |
| **Housing** |  |  |  |
| Households with 1 or more housing problems (cnty), 2014-18 (%) | 24.5 (5.7) | 0.0 |  |
| Housing-insecure (cnty), 2020 (%) | 20.4 (2.9) | 10.6 |  |
| Houses that are owner-occupied (cnty), 2016-20 (%) | 52.4 (6.3) | 0.0 |  |
| Households spending at least half of income on housing (cnty), 2016-20 (%) | 19.9 (3.5) | 0.0 |  |
| Vacant housing (state), 2019 (%) | 9.9 (4.3) | 0.0 |  |
| **Employment** |  |  |  |
| Employed (zipc), 2020 (%) | 57.4 (10.7) | 5.8 |  |
| Mean work hours per week (zipc), 2020 | 38.0 (3.7) | 5.8 |  |
| Government workers (zipc), 2020 (%) | 13.8 (5.3) | 5.8 |  |
| >16 unemployed and seeking work (cnty), 2020 (%) | 10.3 (2.1) | 0.0 |  |
| 16-19 not in school and unemployed (cnty), 2016-20 (%) | 7.4 (2.0) | 0.0 |  |
| **Education** |  |  |  |
| Children in public schools eligible for free/reduced lunch (cnty), 2019-20 (%) | 60.7 (7.5) | 0.0 |  |
| Enrolled in K-12 (zipc), 2020 (%) | 55.6 (19.0) | 5.8 |  |
| 9th grade cohort graduating in 4 years (cnty), 2018-19 (%) | 83.3 (7.4) | 22.1 |  |
| >25 with HS diploma (cnty), 2016-20 (%) | 85.0 (3.7) | 0.0 |  |
| >25 without HS diploma (state), 2019 (%) | 15.2 (3.7) | 0.0 |  |
| Bachelor’s degree or higher (zipc), 2020 (%) | 38.0 (22.4) | 5.8 |  |
| Mean 3rd grade reading test performance (0-10) (cnty), 2018 | 3.0 (0.1) | 17.3 |  |
| Mean 3rd grade math test performance (0-10) (cnty), 2018 | 2.8 (0.1) | 17.3 |  |
| Mean difference between actual and required public school spending per student ($) (cnty), 2019 | -1647.7 (4868.5) | 0.0 |  |
| School racial segregation level relative to local distribution (0.0-1.0) (cnty), 2020-21 | 0.3 (0.1) | 0.0 |  |
| **Residential Environment** |  |  |  |
| County land area (mi2) (zipc), 2020 | 2220.8 (1746.9) | 0.0 |  |
| Racial dissimilarity index^[3]^ (0-100) (cnty), 2016-20 | 61.8 (10.4) | 0.0 |  |
| Childcare centers (per 100K aged <5) (cnty), 2021 | 5.6 (2.2) | 0.0 |  |
| Households with broadband internet connection (cnty), 2016-20 (%) | 85.3 (4.5) | 0.0 |  |
| Avg PM2.5 (cnty), 2018 | 11.9 (2.3) | 0.0 |  |
| Population lacking adequate food access (cnty), 2019 (%) | 11.6 (2.7) | 0.0 |  |
| Population with adequate access to locations for physical activity (cnty), 2020-21 (%) | 93.8 (10.1) | 0.0 |  |
| **Travel and Transportation** |  |  |  |
| Mean commute time to work (min) (zipc), 2020 | 28.4 (6.2) | 5.8 |  |
| Workers driving alone to work for >30 min (cnty), 2016-20 (%) | 45.4 (7.1) | 0.0 |  |
| Workforce driving alone to work (cnty), 2016-20 (%) | 69.1 (8.4) | 0.0 |  |
| Motor vehicle crash death rate (per 100K) (cnty), 2014-20 | 9.0 (2.9) | 0.0 |  |
| Mean major roadway traffic (vol/m) (cnty), 2019 | 1917.9 (1181.4) | 0.0 |  |
| Public transit score (0.0-10.0) (zipc), 2018 | 7.4 (2.9) | 1.0 |  |
| Walkability score (0-100) (zipc), 2022 | 65.3 (26.8) | 1.0 |  |
| **Crime and Substance Use** |  |  |  |
| Number of reported violent crime offenses (per 100K) (cnty), 2014-16 | 688.0 (418.4) | 0.0 |  |
| Number of homicide mortality (per 100K) (cnty), 2014-20 | 14.6 (13.8) | 0.0 |  |
| Delinquency case rate (per 1K minors) (cnty), 2019 | 10.8 (8.4) | 1.0 |  |
| Percent of drinking water violations present (cnty), 2020 | 49.0 | 0.0 |  |
| Age-adj adults reported binge or heavy drinking (cnty), 2019 (%) | 19.1 (2.8) | 0.0 |  |
| **Healthcare Access** |  |  |  |
| Adults with no PCP (state), 2021 (%) | 14.9 (3.2) | 0.0 |  |
| Adults not seen HCP in past year due to cost (state), 2021 (%) | 8.9 (1.2) | 0.0 |  |
| Ratio PCPs to population*1000 (cnty), 2019 | 0.9 (0.2) | 0.0 |  |
| Ratio dentist to population*1000 (cnty), 2020 | 0.9 (0.6) | 0.0 |  |
| Ratio mental health HCPs to population*1000 (cnty), 2021 | 3.8 (1.2) | 0.0 |  |
| Ratio of non-physician PCPs to population*1000 (cnty), 2021 | 1.3 (0.8) | 0.0 |  |
| <65 uninsured (zipc), 2020 (%) | 8.8 (5.6) | 5.8 |  |
| <65 uninsured (cnty), 2019 (%) | 9.8 (2.9) | 1.9 |  |
| Adults <65 uninsured (cnty), 2019 (%) | 12.8 (3.4) | 0.0 |  |
| <19 uninsured (cnty), 2019 (%) | 4.2 (2.0) | 0.0 |  |
| Adults uninsured (state), 2019 (%) | 8.7 (2.2) | 0.0 |  |
| Uninsured (cnty), 2019 (%) | 10.5 (2.8) | 0.0 |  |
| Syringe exchanges (state), 2022 | 21.2 (16.7) | 0.0 |  |
|  | | | |
|  | | | |

| **Table S-2** HIV-related Variables (HIV/AIDS Epidemiology, Transmission routes of HIV, and HIV Criminalization Laws)^[4]^ | | | | |  |  |
| --- | --- | --- | --- | --- | --- | --- |
|  | Mean (SD) or % (SD)  (n=104) | | Missing, % | |  |  |
| **Prevalence** |  | |  | |  |  |
| HIV prevalence (per 100K) (state), 2019 | 424.1 (109.9) | | 0.0 | |  |  |
| HIV prevalence (per 100K) (cnty), 2020 | 830.9 (570.2) | | 9.6 | |  |  |
| HIV prevalence % (cnty), 2020 (%) | 0.8 (0.6) | | 9.6 | |  |  |
| HIV prevalence >13 (per 100K) (cnty), 2019 | 1859.7 (770.9) | | 72.1 | |  |  |
| HIV prevalence >13 % (cnty), 2019 (%) | 1.9 (0.8) | | 72.1 | |  |  |
| **People Living with HIV (PLWH) Demographics** |  | |  | |  |  |
| 13-24 (cnty), 2020 (%) | 2.4 (0.8) | | 10.6 | |  |  |
| 25-34 (cnty), 2020 (%) | 15.3 (2.7) | | 10.6 | |  |  |
| 35-44 (cnty), 2020 (%) | 19.0 (2.2) | | 10.6 | |  |  |
| 45-54 (cnty), 2020 (%) | 24.4 (1.2) | | 10.6 | |  |  |
| >54 (cnty), 2020 (%) | 38.9 (5.6) | | 10.6 | |  |  |
| **HIV Incidence** |  | |  | |  |  |
| Black (cnty), 2020 (%) | 38.3 (25.6) | | 10.6 | |  |  |
| White (cnty), 2020 (%) | 23.2 (9.9) | | 10.6 | |  |  |
| Hispanic (cnty), 2020 (%) | 30.7 (16.5) | | 10.6 | |  |  |
| HIV incidence (per 100K) (state), 2019 | 14.3 (2.2) | | 0.0 | |  |  |
| Estimated HIV incidence (per 100K) (state), 2019^a^ | 12.8 (1.8) | | 0.0 | |  |  |
| HIV incidence (per 100K) (cnty), 2020 | 19.5 (8.1) | | 10.6 | |  |  |
| HIV incidence male (cnty), 2020 (%) | 85.0 (5.0) | | 10.6 | |  |  |
| HIV incidence female (cnty), 2020 (%) | 15.0 (5.0) | | 10.6 | |  |  |
| HIV incidence Black (cnty), 2020 (%) | 41.9 (25.6) | | 10.6 | |  |  |
| HIV incidence White (cnty), 2020 (%) | 17.0 (6.2) | | 10.6 | |  |  |
| HIV incidence Hispanic (cnty), 2020 (%) | 35.5 (18.9) | | 10.6 | |  |  |
| Adult adolescent HIV incidence (per 100K) (state), 2020 | 14.2 (2.3) | | 0.0 | |  |  |
| HIV incidence 13-24 (cnty), 2020 (%) | 19.2 (5.1) | | 10.6 | |  |  |
| HIV incidence 25-34 (cnty), 2020 (%) | 38.1 (2.7) | | 10.6 | |  |  |
| HIV incidence 35-44 (cnty), 2020 (%) | 20.4 (2.4) | | 10.6 | |  |  |
| HIV incidence 45-54 (cnty), 2020 (%) | 12.6 (2.6) | | 10.6 | |  |  |
| HIV incidence >54 (cnty), 2020 (%) | 9.7 (2.4) | | 10.6 | |  |  |
| **Route** **of Transmission (by Gender)** |  | |  | |  |  |
| PLWH male (cnty), 2020 (%) | 82.1 (8.7) | | 10.6 | |  |  |
| PLWH male infected via heterosexual contact (cnty), 2020 (%) | 6.2 (4.5) | | 9.6 | |  |  |
| PLWH male infected via IDU (cnty), 2020 (%) | 8.6 (8.8) | | 7.7 | |  |  |
| PLWH male infected via sex with men (cnty), 2020 (%) | 77.3 (13.6) | | 2.9 | |  |  |
| PLWH male infected via sex with men and IDU (cnty), 2020 (%) | 7.3 (0.9) | | 9.6 | |  |  |
| PLWH male infected through other means (cnty), 2020 (%) | 0.7 (0.3) | | 9.6 | |  |  |
| PLWH female infected via IDU (cnty), 2020 (%) | 23.3 (7.0) | | 9.6 | |  |  |
| PLWH female infected via heterosexual contact (cnty), 2020 (%) | 73.3 (6.9) | | 9.6 | |  |  |
| PLWH female infected via other route (cnty), 2020 (%) | 3.4 (0.4) | | 9.6 | |  |  |
| **AIDS** |  | |  | |  |  |
| AIDS incidence (per 100K) (state), 2019 | 6.3 (1.5) | | 0.0 | |  |  |
| AIDS prevalence (state), 2019 (%) | 0.2 (0.1) | | 0.0 | |  |  |
| **Criminalization** |  | |  | |  |  |
| HIV criminalization laws type (state), 2022 |  | | | 0.0 | | |
| - Sentence Enhancement, % | | 22.1 | |  | | |
| - General Crime Sentencing, % | | 4.8 | |  | | |
| - HIV Law and Gen Crime Sentencing, % | | 17.3 | |  | | |
| - Sentence Enhancement and General Crime Sentencing, % | | 46.2 | |  | | |
| - None, % | | 9.6 | |  | | |
| ^a^ Estimated time of infection using CD4 depletion model from CDC^[5]^ | | | | | |  |

| **Table S-3** *Implementation Outcome Variables for HIV Interventions^[6]^* | | |  |
| --- | --- | --- | --- |
|  | Mean (SD) or % (SD) (n=104) | Missing, % |  |
| **Testing** |  |  |  |
| Ever tested for HIV (cnty), 2016-17 (%) | 47.1 (7.5) | 10.6 |  |
| Tested for HIV, 2016-2017 (cnty), (%) | 15.7 (4.6) | 10.6 |  |
| Knowledge of status (state), 2019 | 80425.7 (50305.9) | 0.0 |  |
| **Linked to Care** |  |  |  |
| Incident HIV - linked to care (state), 2019 | 2233.4 (1372.9) | 0.0 |  |
| Incident HIV - linked to care (cnty), 2020 (%) | 83.7 (3.3) | 10.6 |  |
| **Receiving Care** |  |  |  |
| Receiving care (state), 2019 | 59407.9 (38134.8) | 0.0 |  |
| Receiving care (cnty), 2020 (%) | 71.6 (2.1) | 10.6 |  |
| **Viral Suppression** |  |  |  |
| Number of PLWH with virally suppressed (state), 2019 | 51543.2 (34035.3) | 0.0 |  |
| Virally suppressed (cnty), 2020 (%) | 61.7 (2.1) | 10.6 |  |
| **PrEP** |  |  |  |
| Number of patients prescribed PrEP (state), 2019 | 25016.7 (16323.1) | 0.0 |  |
| PrEP use (cnty), 2020 (%) | 0.2 (0.1) | 10.6 |  |
| PrEP users, female (cnty), 2020 (%) | 6.6 (3.3) | 10.6 |  |
| PrEP users, male (cnty), 2020 (%) | 93.3 (3.3) | 10.6 |  |
| PrEP users 13-24 (cnty), 2020 (%) | 10.4 (2.1) | 10.6 |  |
| PrEP users 25-34 (cnty), 2020 (%) | 41.1 (1.9) | 10.6 |  |
| PrEP users 35-44 (cnty), 2020 (%) | 26.6 (2.3) | 10.6 |  |
| PrEP users 45-54 (cnty), 2020 (%) | 13.1 (1.2) | 10.6 |  |
| PrEP users > 55 (cnty), 2020 (%) | 8.8 (0.9) | 10.6 |  |
| **PrEP Use to HIV Incidence** |  |  |  |
| Ratio of 2021 PrEP users to 2020 HIV incidence (cnty), 2020 | 12.9 (4.4) | 10.6 |  |
| Ratio of 2021 female PrEP users to 2020 female HIV incidence (cnty), 2021 | 5.2 (1.3) | 10.6 |  |
| Ratio of 2021 male PrEP users to 2020 HIV incidence (cnty), 2021 | 14.1 (4.8) | 10.6 |  |
| Ratio of 2021 25-34 PrEP users to 2020 HIV incidence (cnty), 2020 | 13.7 (4.6) | 10.6 |  |
| Ratio of 2021 35-44 PrEP users to 2020 HIV incidence (cnty), 2020 | 17.6 (7.6) | 10.6 |  |
| Ratio of 2021 45-54 PrEP users to 2020 HIV incidence (cnty), 2020 | 13.9 (6.0) | 10.6 |  |
| Ratio of 2021 >54 PrEP users to 2020 HIV incidence (cnty), 2020 | 12.8 (5.1) | 10.6 |  |
| **Mortality** |  |  |  |
| HIV mortality rate (per 100K) (state), 2019^a^ | 6.6 (2.5) | 0.0 |  |
| Calculated HIV mortality rate (per 100K) (state), 2020 ^a^ | 6.4 (2.2) | 0.0 |  |
| AIDS mortality rate (per 100K) (state), 2019 ^a^ | 5.0 (1.8) | 0.0 |  |
|  | | | |
| ^a^ Mortality among those who are HIV+ and with AIDS (respectively) and are not necessarily attributed to HIV/AIDS-related causes | | | |

1. Values are mean (SD) or median (Q25, Q75) for continuous variables; percentages or counts or both for categorical variables. Values of polytomous variables may not sum to 100 percent due to rounding.
2. Organizations enumerated include business, labor, political, professional, athletic, civic, volunteer, and religious organizations. This variable can be used to assess the level of community engagement, social cohesion, and availability of opportunities for participation in social organizations within a population.
3. The dissimilarity index used here measures the extent to which the county is segregated by black/white races. Higher values indicate increased segregation within the county. This index ranges from 0 to 100 (to express the Dissimilarity Index as a percentage, multiply it by 100). A score of 0% denotes no segregation, while 100% signifies complete segregation.
4. Values are mean (SD) or median (Q25, Q75) for continuous variables; percentages or counts or both for categorical variables. Values of polytomous variables may not sum to 100 percent due to rounding.
5. Song, Ruiguang et al. “Using CD4 Data to Estimate HIV Incidence, Prevalence, and Percent of Undiagnosed Infections in the United States.” Journal of acquired immune deficiency syndromes (1999) vol. 74,1 (2017): 3-9. doi:10.1097/QAI.0000000000001151
6. Values are mean (SD) or median (Q25, Q75) for continuous variables; percentages or counts or both for categorical variables.
